# Patterns of Joint Improvisation in Adults with Autism Spectrum Disorder

**DOI:** 10.3389/fpsyg.2017.01790

**Published:** 2017-10-24

**Authors:** Rachel-Shlomit Brezis, Lior Noy, Tali Alony, Rachel Gotlieb, Rachel Cohen, Yulia Golland, Nava Levit-Binnun

**Affiliations:** ^1^Sagol Center for Brain and Mind, Baruch Ivcher School of Psychology, Interdisciplinary Center Herzliya, Herzliya, Israel; ^2^Theatre Lab, Weizmann Institute of Science, Rehovot, Israel

**Keywords:** joint improvisation, autism spectrum disorder, motion synchronization, motor skills, social skills

## Abstract

Recent research on autism spectrum disorders (ASDs) suggests that individuals with autism may have a basic deficit in synchronizing with others, and that this difficulty may lead to more complex social and communicative deficits. Here, we examined synchronization during an open-ended joint improvisation (JI) paradigm, called the mirror game (MG). In the MG, two players take turns leading, following, and jointly improvising motion using two handles set on parallel tracks, while their motion tracks are recorded with high temporal and spatial resolution. A series of previous studies have shown that players in the MG attain moments of highly synchronized co-confident (CC) motion, in which there is no typical kinematic pattern of leader and reactive follower. It has been suggested that during these moments players act as a coupled unit and feel high levels of connectedness. Here, we aimed to assess whether participants with ASD are capable of attaining CC, and whether their MG performance relates to broader motor and social skills. We found that participants with ASD (*n* = 34) can indeed attain CC moments when playing with an expert improviser, though their performance was attenuated in several ways, compared to typically developing (TD) participants (*n* = 35). Specifically, ASD participants had lower rates of CC, compared with TD participants, which was most pronounced during the following rounds. In addition, the duration of their CC segments was shorter, across all rounds. When controlling for participants’ motor skills (both on the MG console, and more broadly) some of the variability in MG performance was explained, but group differences remained. ASD participants’ alexithymia further correlated with their difficulty following another’s lead; though other social skills did not relate to MG performance. Participants’ subjective reports of the game suggest that other cognitive and emotional factors, such as attention, motivation, and reward-processing, which were not directly measured in the experiment, may impact their performance. Together, these results show that ASD participants can attain moments of high motor synchronization with an expert improviser, even during an open-ended task. Future studies should examine the ways in which these skills may be further harnessed in clinical settings.

## Introduction

Autism spectrum disorder (ASD) is a neurodevelopmental disorder that affects a growing number of individuals worldwide (Elsabbagh et al., 2012), and is characterized by impairments in social and communicative skills, and restricted and repetitive behaviors (American Psychiatric Association, 2013). Interestingly, a new wave of research in autism is confirming what decades of reports from individuals with autism and their caregivers have suggested – that autism is characterized by fundamental differences in sensory-motor functioning (Bluestone, 2005; Fournier et al., 2010; Torres et al., 2012; Lloyd et al., 2013). Several authors have recently suggested that these altered ways of perceiving and moving in the world (alone, as well as with other people) have important downstream implications for many aspects of social functioning in autism, including imitation, joint attention, collaboration, and communication (Dowd et al., 2010; De Jaegher, 2013; Levit-Binnun et al., 2013; Marsh et al., 2013). Yet studies of sensory-motor abilities in ASD have thus far mostly focused on individuals’ isolated functioning, such as hyper- and hypo-sensitivities, or difficulty coordinating perception and action (Fournier et al., 2010; Torres et al., 2012). Thus, the relation between individual motor abilities, interpersonal motor abilities, and broader social skills in ASD remains unclear.

Recent research suggests that sensory-motor synchronization between individuals might play a crucial role in successful social interactions (Kokal et al., 2011). Developmental studies have demonstrated that infants exhibit profound patterns of sensory-motor synchronization with their caregivers, and that such patterns are associated with further developmental consequences (Feldman, 2007). Along similar lines, healthy individuals engaged in interaction have been shown to spontaneously and subconsciously mimic and synchronize their expressions, vocalizations, and movements (Marsh et al., 2009). Importantly, both spontaneous and induced synchronization have been shown to have a powerful impact on the quality of social interaction (van Baaren et al., 2004; Valdesolo et al., 2010; Chartrand and Lakin, 2013). By contrast, reduced interpersonal motor synchronization has been found in individuals with schizophrenia (Varlet et al., 2012; Lavelle et al., 2014), and social anxiety disorder (Varlet et al., 2014).

Decades of research have shown that individuals with autism have deficits in imitating others’ gestures, facial expressions, and vocalizations (Edwards, 2014; Vivanti and Hamilton, 2014), and interventions that teach children with autism to imitate have been shown to affect development in language, play skills, and joint attention (Ingersoll, 2008). Accordingly, recent theoretical models of autism suggest that sensory-motor abnormalities, such as in imitation, might directly underlie the ability of individuals with autism to become socially connected with others (Fournier et al., 2010; Gowen and Hamilton, 2013; Marsh et al., 2013; Behrends et al., 2016). Importantly, however, studies of imitation in ASD have largely focused on the content, rather than the timing, of behavior, and have mostly relied on subjective observational methods that allow only for gross analyses of movement and its content. Since successful communication relies not only on *what* is transmitted, but also on *when*, subtle timing differences in autism may contribute to the breakdown of communication, above and beyond problems with transmitted content (Fitzpatrick et al., 2016).

Recent emergent research, using techniques drawn from dynamical systems analysis, has been showing that individuals with autism have reduced interpersonal synchronization. For instance, Marsh et al. (2013) have shown that while typical children sitting alongside their parents on separate rocking chairs will automatically synchronize their motions, children with autism lack this automatic tendency. Furthermore, children with ASD engaged in a synchronous drumming task with an experimenter synchronized less than typically developing (TD) participants, on certain measures (Fitzpatrick et al., 2013; Romero et al., 2016). And adolescents with ASD demonstrated less synchronization with their parents on a pendulum-synchronization task during both spontaneous and intentional coordination (Fitzpatrick et al., 2016). Further, the ability of individuals with autism to engage in synchronous tapping with a computerized interface (disguised as a human collaborator), has been shown to correlate with higher cognitive empathy (Koehne et al., 2016b). Finally, adults with ASD show reduced ability to modulate their grasping action when coordinating their motion with another player (Curioni et al., 2017). These studies provide initial evidence for reduced temporal synchronization in social situations in individuals with autism, and suggest that synchronization may be significantly associated with their social skills. Importantly, however, these studies have thus far focused on rhythmic, repetitive, synchronization, which has limited ecological validity in modeling the complexities of real-life, open-ended interactions.

In the current study, we aim to use an innovative experimental setup for studying open-ended interactions, the mirror game (MG). The MG enables high-resolution motion measurements from two players engaged in a simple joint improvisational (JI) game (Noy et al., 2011). In the first part of the study, we aim to use the MG to determine whether individuals with autism can attain moments of highly synchronized motion when playing with an expert improviser. Second, we aim to determine whether their expected difficulties with synchronization can be explained by their basic motor difficulties, and whether they correlate with their everyday social skills.

### Enabling and Measuring Joint Improvisation: The Mirror Game Paradigm

**FIGURE 1:**
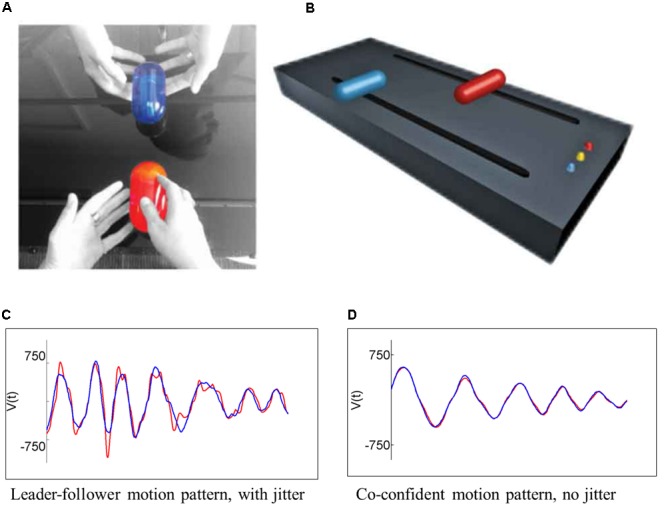
In the mirror game paradigm, two players are instructed to “create movements together, like a conversation in movements,” holding two handles on two parallel tracks **(A)** taking turns leading, following, or jointly improvising (with no designated leader). **(B)** Their movements are sampled at 50 Hz and at a spatial resolution of 0.94 mm. The majority of the time, players display a **(C)** leader–follower pattern, in which the follower (red) shows ‘jitter’ corrective motions around the leader’s (blue) smooth trajectory. Players can also attain moments of **(D)** co-confident pattern, in which the two players have highly synchronized smooth motion tracks, with no jitter, indicative of the fact that they are moving as a coupled unit rather than reactively.

The MG is based on a traditional practice in improvisational theater and dance, in which two players imitate each other, with the aim of enhancing interpersonal connection (Schechner, 1994). Noy et al. (2011) reduced the MG to one-dimensional linear motion, and measured the hand motions of two people mirroring each other while moving handles along parallel tracks, at high temporal and spatial resolution (**Figures 1A,B**). Players were instructed to “create synchronized and interesting motions,” taking turns following or jointly improvising (with no designated leader). Rather than using repetitive motion patterns, this setup enables players to move freely, creating movements that are fast or slow, long, or short, with or without a repetitive sequence, as they choose.

Using fine-grained kinematic analysis of the motion strokes, Noy et al. (2011) identified two distinctive patterns of motion coordination. During times of distinct leader–follower roles, the leader’s motion was smooth, whereas the follower showed a characteristic 1–2 Hz oscillation, or jitter, around the leader’s confident trajectory (**Figure 1C**), indicative of the follower’s attempts to predict the leader’s motion (Noy et al., 2015a). By contrast, expert improvisers were capable of attaining moments of co-confident (CC) motion, in which both players displayed smooth, jitter-less, motion patterns (indicative of an internally driven, rather than reactive motor plan; **Figure 1D**). Notably, in these moments of CC motion, players’ movements were highly synchronized, at less than 180 ms apart (i.e., too fast to be controlled by visual feedback alone). It was suggested, and corroborated by a corresponding mathematical model, that in these special moments of CC motion the two players act as a *coupled unit* or as two leaders in agreement, *co-creating* a motion rather than settling on a reactive leader–follower pattern (Noy et al., 2011). A recent study demonstrated that CC motion in the MG is associated with increased heart rate, and a subjective feeling of togetherness between the players (Noy et al., 2015b). Further studies have demonstrated that novice players can also attain CC motion patterns when playing with an expert improviser (Hart et al., 2014), and that this ability varies with adult attachment styles (Feniger-Schaal et al., 2016) (see Supplementary Table 1 for selected data from this latter study).

The MG paradigm has been taken up by several research groups, which have shown that players who share similar individual motion patterns show greater synchronization (Słowiñski et al., 2016); and that when played against an avatar, synchronization is improved when the avatar is more attractive (Zhao et al., 2015), and when predictive gaze cues are available (Khoramshahi et al., 2016). Moreover, Słowiñski et al. (2017) have used the MG to characterize the different motion patterns of patients with schizophrenia, and to create a highly predictive diagnostic classifier. In sum, the MG setup enables a rich investigation of the interpersonal motion characteristics during JI. Here, we apply this paradigm, for the first time, to the patterns of JI in individuals with autism, and relate these to their broader social and motor skills.

### Aims and Hypotheses

The study aimed to examine the following questions:

(1) What is the pattern of JI in participants with ASD? Specifically, can they attain CC motion in the MG? And if so, does their CC motion differ from TDs’ (on motion parameters such as complexity, speed, and CC duration)?
(2) Are MG motion parameters impacted by participants’ motor skill and social skills?
(3) What is the participants’ experience of the game?

We hypothesized that given their difficulty with synchronization (Marsh et al., 2013; Fitzpatrick et al., 2016), ASD participants would have lower, or non-existent CC rates, and that their CC duration would be briefer. Further, given their tendency for repetitive motion (American Psychiatric Association, 2013) we hypothesized that their motion would be less complex. Given that individual motor skills are necessary for interpersonal motor coordination, we hypothesized that participants’ motor skills would predict MG motion parameters; and given the literature linking synchronization and social abilities in the TD population, we hypothesized that ASD participants’ ability to synchronize on the MG would correlate with their social skills (De Jaegher, 2013; Peper et al., 2016). All other analyses were exploratory in nature, and aimed to provide a richer picture of JI in ASD.

## Materials and Methods

### Participants

Thirty-four participants with high-functioning ASD and 38 TD participants were recruited for the study. Participants with ASD were recruited from the Beit Ekstein organization group homes, from Ariel University, and through advertisements in various autism organizations. Diagnosis for ASD participants was confirmed using the Autism Diagnostic Observation Schedule (ADOS) (Lord et al., 2012). TD participants were recruited from among the Interdisciplinary Center Herzliya (IDC) student population and staff, and through word of mouth. These were matched in age, gender, and IQ with the ASD participants (see **Table 1**). Three TD participants reported having a prior psychiatric diagnosis, and were therefore excluded from the study. The experimental procedures were approved by the ethical review board of the IDC and the Israel Ministry of Social Welfare. Informed consent was obtained from all participants and/or their legal guardians.

**Table 1:** Participants’ background characteristics.

	ASD (*n* = 34) Mean (range)	TD (*n* = 35) Mean (range)	Statistic (df)	*p*-value
Gender	91% male	80% male	χ^2^ = 1.38 (1)	0.31
Age	28.6 (20–45)	25.9 (19–45)	*t* = 1.75 (67)	0.09
Years of education	13.0 (12–18)	14.0 (12–18)	*t* = 2.08 (66)	0.04
Performance IQ (WASI)	11.6 (5–22)	13.5 (6–24)	*t* = 2.03 (67)	0.05
Verbal IQ (WASI)	12.4 (4–18)	13.4 (5–19)	*t* = 1.16 (67)	0.25
Toronto Alexithymia Scale	52.9 (28–75)	41.4 (24–65)	*t* = 4.29 (65)	<0.001
Toronto Empathy Questionnaire	39.1 (25–50)	50.4 (35–63)	*t* = 7.06 (67)	<0.001
Reading the Mind in the Eyes	22.3 (10–32)	26.0 (13–34)	*t* = 2.73 (67)	<0.001
Social Responsiveness Scale – Self-report	63.2 (52–77)	49.9 (36–75)	*t* = 6.21 (67)	<0.001
Social Responsiveness Scale – Parent/caregiver	62.3 (41–76)	–	–	–
Repetitive Behaviors Scale – Parent/caregiver	8.4 (0–25)			

### Procedures

The experimental sessions lasted 2–3 h and took place at the Interdisciplinary Center Herzliya. In order to facilitate the experimental procedures for ASD participants, whenever possible (*n* = 29) the experimental session was split in two sessions, with procedures that did not require a lab (e.g., questionnaires) conducted in the participant’s home environment.

#### Background Characteristics

Autism symptoms in all participants were assessed using the adult self-report version of the Social Responsiveness Scale (SRS) (Constantino, 2012). For ASD participants, parents or caregivers completed the parent/caregiver version of the SRS (Constantino, 2012), and the Repetitive Behaviors Scale (Lam and Aman, 2007), providing an additional view on their everyday social and behavioral skills. Diagnosis for ASD participants was confirmed using the ADOS (Lord et al., 2012). All participants also completed a background questionnaire, detailing their experience in drama, dance, JI, and other forms of movement, and providing details on any psychiatric diagnosis and medication they regularly take. To gain indicators of IQ, two sub-scales of the Hebrew version of the Wechsler Abbreviated Scale of Intelligence (WASI) (Wechsler, 1998) were administered – Vocabulary and Matrices – and served as indicators of Verbal and Performance IQ, respectively (as used in the English WASI; Wechsler and Hsiao-pin, 2011). Standardized scores of each sub-scale are reported here (see **Table 1**).

#### The Mirror Game Procedure

In the MG setup, participants played against an expert improviser (RSB) who has 13 years of experience in contact improvisation and 10 years of experience in working with individuals with autism. Players faced each other holding handles which can move along parallel tracks (**Figures 1A,B**). Players were told that this is a collaborative game whose purpose is to “create movements together like a conversation of movements.” The motion of the two handles was sampled at 50 Hz. The game comprised of three rounds lasting 3 min each, signaled by a bell sound. In the first round, the participant led (Leading round), in the second the participant followed the experimenter’s lead (Following round), and in the third round the two players created motions together, “with no leader or follower” (JI round). Rounds were separated by 10 s periods of rest and participants were instructed not to talk during the game.

##### Participants’ experience of the game

Following each session, participants provided a rating for which round was easiest and hardest, and answered an open question regarding their experience of the game. Participants then completed a brief post-game questionnaire regarding their affective experience and their subjective view of their partner’s responsivity (see Supplementary Materials Section 1.2 “Post-game questionnaire”). Due to technical reasons, data from only a subset of 27 ASD and 9 TD participants are available.

#### Motor Skills

Motor abilities were assessed using both general and specialized tasks intended to tease apart the motor skills necessary for successfully synchronizing with another player on the MG.

General motor tasks included:

(a) The Revised Neurological Examination for Subtle Signs (PANESS) for basic motor and coordination abilities (Denckla, 1985). The PANESS test provides sum scores for six sub-task (e.g., number of hand-taps, or number of hops on one leg in a given time) which vary from task to task, as well as correct/incorrect scores for 12 of the sub-tasks (e.g., did the participant switch legs while hopping, though instructed to stay on one foot). To enable a comparison of scores across participants, a composite score for the PANESS sub-tasks was computed as the average of *z*-scores in all continuously counted sub-tasks, multiplied by the percent of correctly performed tasks.

(b) The Florida Apraxia Battery to assess complex motor planning and dyspraxia (Gonzales et al., 1997). The dyspraxia score was computed as percent correct of 10 trials (e.g., “show me how you would brush your hair”).

(c) An imitation battery based on Fitzpatrick et al. (2013), in which participants were instructed to imitate five sequences of three motions performed by the experimenter, either with or without an object, on their body, or in space. Participants were given a full two points per trial, if they imitated the experimenter with the correct actions, in the correct sequence. The sum score of the imitation task was computed as the simple sum of all trial scores.

These three general motor tasks were videotaped and scored by two raters blind to diagnosis. Inter-rater reliability was kappa > 0.8 for all measures. The dyspraxia score was computed as percent correct trials, and in the imitation task, participants were given.

**FIGURE 2:**
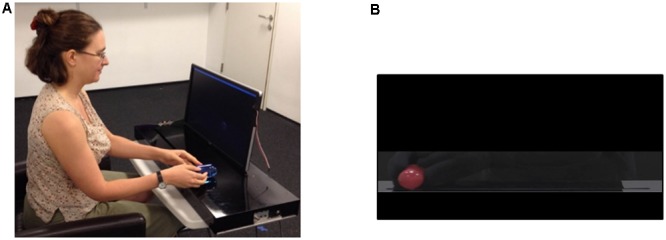
Experimental setup for the specially designed motor tasks. **(A)** Participants sat in front of the mirror game console, with a screen fitted to the track farther from them. **(B)** A static or moving handle was displayed on the screen, and they were instructed to follow it.

For the battery of specially designed motor tasks, the participant sat in front of the MG console, holding the blue handle in the track closest to them. A screen fitted to the second, more distant, track, displayed static and video images of a pre-recorded red handle (**Figure 2**).

In the *Reaching task* (based on: Glazebrook et al., 2008), participants viewed 15 static images of the red handle in six different positions displayed for 4 s at a time, and were instructed to “move your handle across from the red handle’s position as fast and as accurately as you can using your dominant hand.” A measure of accuracy was computed as the distance (in mm) between the target and the participants’ handle after they stopped.

In the *Proprioception task*, intended to measure participants’ ability to sense their own body’s position in space, the same stimuli were used, except each image was only displayed for 2 s, followed by a black screen for 4 s. Participants were instructed to “close your eyes once you see the image disappear, and try to move your handle to the position in which you think you saw the red handle.” To ensure that participants cannot see their own hand, the experimenter simultaneously placed a black veil in front of the participants’ eyes. An accuracy measure was extracted as described above.

The *Repetitive Motion task* was intended to measure participants’ maximum motion speed. Two sponges were fitted to each extremity of the MG track closest to the participant, and participants were instructed to “move the handle back and forth as fast as possible from one end of the track to the other.” They started with one 20 s round with their dominant hand, then had a 10 s pause, and then continued for a second round of 20 s with their non-dominant hand. A measure of the average maximum velocity per segment, across both rounds, was computed.

Finally, in the *Following a Moving Target task* an MG ‘Following’ round was simulated. Participants viewed a 3 min video of a red handle moving in typical MG motions, and were instructed to “follow the red handle as best as you can.” This video was created by filming the same expert improviser who played against the participants in the MG moving the handle as though they were ‘leading,’ and then masking all traces of their hands and body. %CC was computed as a measure of synchronization. Due to technical reasons data from only 22 ASD and 9 TD participants was available for this task.

#### Social Skills

Participants completed a set of questionnaires assessing empathic tendency (Toronto Empathy Questionnaire, TEQ; Spreng et al., 2009), emotional recognition (Reading the Mind in the Eyes, RMET; Baron-Cohen et al., 2001), and ability to recognize one’s own emotions (Toronto Alexithymia Scale, TAS; Bagby et al., 1994).

Following the MG, participants completed a *Naturalistic Conversation task*. Participants were instructed to speak freely with the experimenter for 5 min about a given topic (e.g., a recent vacation). Conversations were videotaped and rated by two coders blind to the study groups for the number of looks, headshakes/nods and smiles, and for global ratings of affective engagement and flow (García-Pérez et al., 2007). Affective engagement was defined as the degree of emotional connectedness between the participant and the experimenter, and ranged from a rating of 1 (no emotional connection between partners) to 5 (strong emotional connection between partners, as exhibited in high positive affect directed between the partners, and participants’ comfort). Flow was defined as the degree of smooth exchanges between the participant and the experimenter, and ranged from a rating of 1 (minimum exchange) to 5 (very smooth exchange, with a steady back-and-forth dialog; neither interlocutor is talking more than the other). Inter-rater reliability was kappa > 0.8 for all variables. Given that the continuous variables extracted from the videos (looks, headshakes, and smiles), were positively correlated, we computed a composite score of conversation skills as the average of *z*-scores of all three variables.

### Analysis

Prior to statistical analyses, data were inspected visually, and the Kolmogorov-Smirnoff test of normality was performed. Where a comparison of variables was planned (e.g., *t*-test), Levene’s test of homogeneity of variance was first performed, and results reported accordingly. If data did not meet assumptions of normality, alternative statistical tests were used, as detailed below.

#### Background Data

To determine whether participants differed in their prior experience with drama, dance, and other forms of JI, a series of Mann–Whitney *U* tests between the ASD and TD groups were performed.

#### Motor Traces Analysis

First, we examined participants’ basic motor characteristics in the MG. To determine whether ASD participants’ motion complexity was lower than ASD participants’, we computed a measure of complexity based on wavelet analysis, which has been previously used to describe complexity in the MG (Noy et al., 2011)^1^. To determine whether participants differed in their motion speed, a measure of velocity was computed by averaging the maximum segment velocity across each game round. These dependent variables were entered into 2 (ASD and TD) × 3 (Leading, Following, and JI) repeated-measures ANOVA analyses in turn. *Post hoc* tests were computed where relevant.

To examine the dyadic motion characteristics, we used the previously developed notion of CC motion, defined as periods of high synchrony with little jitter (Noy et al., 2011), using an updated algorithm (for details, see Noy et al., 2015a,b)^2^. Data were parsed into motion segments, defined as periods of motion between two zero velocity events longer than 0.2 s and shorter than 8 s. Segments were considered as part of a CC period if they matched five conditions: (1) they contained exactly one acceleration zero crossing (that is, a single velocity peak, with no jitter); (2) the temporal distances of their stopping-point events (dT) were shorter than 0.09 s; (3) the normalized velocity error (dV) between the two segments was smaller than 0.95; the distance in velocity peaks was smaller than 0.3; and (5) the two players’ motion was in the same direction (see Supplementary Material Figure 1). Our main variable of interest was a measure of the % of CC segments in each round. We further computed the average CC duration (sec) per participant, per round. Once again, we entered these dependent variables into 2 (ASD and TD) × 3 (Leading, Following, and JI) repeated-measures ANOVA analyse (Given that %CC has a negative binomial distribution, we repeated the same analyses using negative binomial analyses. As these yielded similar results, we report only the ANOVA results here, for simplicity).

Given that CC is dependent on velocity (Noy et al., 2017), and having found that ASD participants moved more slowly in the Following round, we sought to determine whether the reduced velocity among ASD participants led them to less CC, compared with TD participants, or whether their ability to attain CC in the Following round was independent. We proceeded with exploratory analyses, in which we compared the %CC under different velocity ranges (specifically, we divided the range between 0 and 2000 mm/s range, which comprised 97% of the data, into four equal bins). To determine the relative effect of velocity on CC in each group, we computed a 4 (bins) × 2 (ASD and TD) repeated-measures ANOVA analysis. Given the fact that several participants did not play in all velocity ranges, data from 24 ASD and 33 TD participants was included in the analysis. For this same reason, the data for the Leading and JI rounds were deemed insufficient (*n* < 14), and analyses were not performed.

Finally, to determine whether the %CC levels we found in both the ASD and TD groups could be deemed above chance, we conducted a pseudo-pairs analysis. For each participant a pseudo-pair was created by matching his/her position vector with a position vector of another participant (not the expert improviser, who was the actual partner). The matching was done between the same rounds (e.g., round 1 of player <i> is matched with round 1 of player <j>, and so forth). We repeated this procedure for all possible pair of rounds in the TD group (resulting in 1,683 pseudo-pairs) and all possible pair of rounds in the ASD group (resulting in 1,785 pseudo-pairs). We then compared these pseudo data with our actual data using a series of independent *t*-test.

#### Relating the MG Performance to Motor and Social Skills

To determine whether participants differed in their motor and social skills, we computed independent *t*-tests on all motor and social measures. To determine the impact of motor and social skills on the ability to attain CC, we performed two separate binomial regression analyses, using motor and social predictors in turn, and focusing on the %CC in Following as our dependent variable of interest. We chose as predictors those variables which were significantly different between groups, in order to better refine the search for motor or social variables which would best predict CC ability (see detailed description below). As a preliminary test, we ran these analyses using the average CC as a dependent measure, but did not find any significant predictors; therefore, we focus our report on the %CC in the Following round.

#### Relating the MG Performance to Subjective Experience

To determine whether ASD participants differed from TD participants in their affective experience of the game (post-game questionnaire), we compared the two groups’ responses using Mann–Whitney *U* tests.

To determine whether ASD and TD participants perceived the difficulty of rounds differently, we performed a chi-squared test, comparing the distribution of ratings across the two groups for easiest, and then hardest round. To determine whether participants’ ratings of hardest round related to MG performance, we ran three binomial regression models, with %CC in each round of the MG as a dependent variable by turn, and the participants’ report of hardest round as categorical predictor; separately for ASD and TD groups. We hypothesized an effect of round, such that participants who rated a particular round as most difficult would have lower %CC on this particular round compared to the other participants.

Given the small number of TD responses available, we proceeded with exploratory analyses in the ASD group only. To determine whether participants’ affective ratings correlated with MG performance, we computed Spearman’s correlations with %CC in each round. Participants’ open-ended explanations for which round they found easiest or hardest were examined qualitatively, to determine the main themes in participants’ descriptions.

#### Preliminary Analyses

To determine the effect of group differences in verbal IQ and years of education on our parameters of interest, we re-ran all of the analyses entering these as covariates. The effect of these variables was not statistically significant (*p* > 0.19) and the pattern of results remained the same, therefore we focus only on the main variables of interest henceforth. Given the possible impact of IQ on performance in the MG, as a further check we computed Pearson correlations between our main dependent variable, %CC in each round, and both PIQ and VIQ. There was no significant correlation in either of the groups (see Supplementary Tables 2, 3), suggesting that joint synchronization did not vary with IQ.

Furthermore, despite the fact that all participants’ diagnosis was confirmed on the ADOS, several participants scored below the cutoff for autism on the SRS (either by participant or caregiver report). As a stringent check, we re-ran the analyses excluding those ASD participants who scored below the cutoff, but did not find any significant qualitative changes in results. Thus, we proceed to report on the entire sample.

## Results

### Participants’ Background Characteristics

ASD and TD groups did not differ significantly in their experience of drama (*U* = 577, *p* = 1.00), dance (*U* = 615, *p* = 0.053), experience with JI (*U* = 661, *p* = 0.192), and familiarity with the MG (*U* = 553, *p* = 0.676). ASD participants reported taking anti-psychotic medication (*n* = 2), anti-depressants (*n* = 4); stimulants (*n* = 2), and anti-epileptic medication (*n* = 1); TD participants reported taking stimulants (*n* = 3), anti-depressants (1), anti-epileptic drugs (1), and medication for other medical conditions (e.g., stomach acid, asthma). Two ASD participants self-reported having a comorbid disorder, one with OCD, another with anxiety disorder. Preliminary analyses revealed that inclusion or exclusion of participants who reported taking medications did not change the effects and directionality of the results; hence, we included these participants in all reported analyses.

### Basic Motor Characteristics in the MG

First, we characterized individual motor characteristics, i.e., motion complexity and average velocity within each of the three MG rounds, and compared them between ASD and TD groups (see **Table 2**).

**Table 2:** Participants’ motor measures in the Mirror Game: complexity, velocity, percent CC, and CC duration Mean (SD).

Motor measure	MG round	ASD	TD
Complexity (wavelets	Leading	0.030 (0.013)	0.026 (0.013)
decomposition	Following	0.030 (0.005)	0.030 (0.004)
compression ratio)	Joint improvisation	0.017 (0.007)	0.014 (0.007)

Velocity (mm/s)	Leading	852.3 (352.4)	691.9 (335.2)
	Following	806.5 (172.4)	899.4 (105.2)
	Joint improvisation	609.7 (281.6)	494.0 (231.5)

Percent co-confident	Leading	9.8 (14.7)	10.9 (12.8)
(CC) motion	Following	8.2 (6.7)	25.7 (10.9)
	Joint improvisation	10.3 (13.0)	12.5 (13.9)

CC duration (s)	Leading	1.99 (1.6)	2.19 (1.2)
	Following	2.05 (1.2)	3.78 (1.4)
	Joint improvisation	3.10 (3.5)	4.6 (4.5)

**FIGURE 3:**
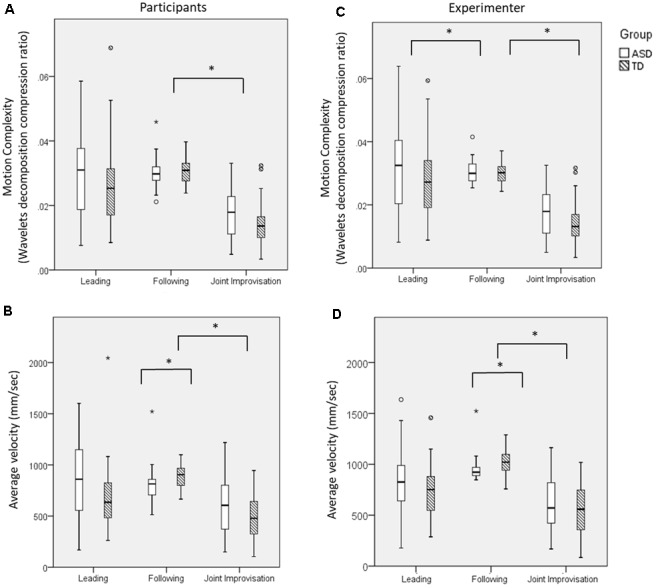
Basic motor characteristics in the MG. **(A)** Participants’ motion complexity differed significantly across rounds, but did not differ between groups. **(B)** Participants’ motion velocity was slower for all participants during JI, compared with Leading or Following rounds. ASD participants moved more slowly when following an expert improviser. **(C)** The experimenter’s motion complexity did not differ significantly across both groups within each round, though there was a significant effect of round which mirrored the motion patterns of participants. **(D)** The experimenter’s average velocity similarly differed across rounds, mirroring the motion patterns of participants; the experimenter’s motion was faster when playing with the TD participants, but did not differ across groups in other rounds. Large asterisks represent *p* < 0.05 group differences. Boxplots: the solid line represents the median of the distribution; the outlines of the box represent the interquartile range, or 25th–75th percentiles; the whiskers represent the upper and lower quartiles, excluding outliers; small points and asterisks represent outliers and extreme outliers, respectively.

Motion complexity differed across rounds [main effect of round: *F*(2,130) = 53.93, *p* < 0.001; see **Figure 3A**], being lower in the JI round compared with the Leading [*t*(66) = 7.23, *p* < 0.001] and Following [*t*(66) = 13.93, *p* < 0.001] rounds, which were not different from each other [*t*(67) = 1.20, *p* = 0.23]. Participants with ASD did not differ from TD participants in their motion complexity [no main effect of Group: *F*(1,65) = 2.13, *p* = 0.15 nor Group × Round interaction *F*(2,130) = 1.16, *p* = 0.32].

Motion velocity (**Figure 3B**) was different across rounds [main effect of round: *F*(2,130) = 24.61, *p* < 0.001]. *Post hoc* analyses showed that all participants were slower in the JI round compared with the Leading [*t*(66) = 4.24, *p* < 0.001] and Following rounds [*t*(66) = 8.52, *p* < 0.001]; and marginally slower in the Leading, compared with Following rounds [*t*(67) = 1.70, *p* = 0.09]. While no significant main effect of Group [*F*(1,65) = 1.54, *p* = 0.22] was found, ASD and TD participants differed significantly by interaction of Round and Group [*F*(2,130) = 5.35, *p* < 0.01]. TD participants moved significantly faster than ASD participants when Following [*t*(66) = 2.68, *p* < 0.001]; while ASD participants moved marginally faster than TD participants in the Leading [*t*(67) = 1.94, *p* = 0.057] and JI [*t*(65) = 1.84, *p* = 0.071] rounds.

Further, we examined whether group differences reported above could stem from different performance of the expert improviser when playing with ASD and TD participants (**Figures 3C,D**). The expert improviser’s motion velocity did not differ by Group [*F*(1,65) = 0.337, *p* = 0.564], nor by an interaction of Round and Group [*F*(2,130) = 3.47, *p* = 0.07]. *Post hoc* between-group tests revealed that the experimenter’s velocity did not differ when playing with ASD and TD participants in the Leading or JI rounds [Leading, *t*(67) = 1.38, *p* = 0.17; JI, *t*(65) = 1.17, *p* = 0.25]; but her velocity was faster when playing with TD participants, compared with ASD participants, when they followed her [*t*(66) = -3.12, *p* = 0.003]. The expert improviser’s motion complexity did not differ by Group [*F*(2,130) = 0.49, *p* = 0.61]; and there was no significant interaction effect of Round and Group [*F*(2,130) = 2.51, *p* = 0.12; see **Figure 3C**). *Post hoc* between-group tests corroborated this pattern [Leading, *t*(67) = 0.96, *p* = 0.34; Following, *t*(66) = 0.43, *p* = 0.67; JI, *t*(65) = 1.58, *p* = 0.12].

To summarize, we found that ASD and TD participants did not differ significantly in their motion complexity; nor did the expert improviser’s motion differ significantly when playing with either ASD or TD participants. However, ASD and TD participants showed different patterns of motion velocity: while TD participants moved faster than ASD participants in following, their motions were slower than ASD participants’ in the Leading and JI rounds.

### Participants with ASD Can Attain CC, But Show Attenuated Levels of CC When Following, and Shorter CC Periods across All Game Rounds, Compared with TD Participants

As a second step, we identified and quantified the amount and duration of highly synchronized CC motion, during the three MG rounds and compared them between groups (see **Table 2**).

**FIGURE 4:**
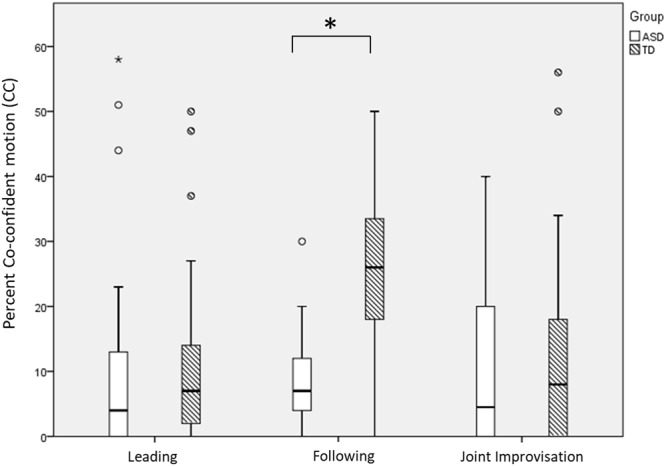
Percent co-confident (CC) motion by game round and group. Participants with ASD had lower CC rates than TD participants (main effect of Group), and this was most pronounced in the Following round (interaction effect of Group and Round). Large asterisks represent *p* < 0.05 group differences. Boxplots: the solid line represents the median of the distribution; the outlines of the box represent the interquartile range, or 25th–75th percentiles; the whiskers represent the upper and lower quartiles, excluding outliers; small points and asterisks represent outliers and extreme outliers, respectively.

Participants differed in their percent CC attained by a main effect of Round [*F*(2,67) = 6.60, *p* = 0.002], a main effect of Group [*F*(1,67) = 13.09, *p* = 0.001] and an interaction effect of Round and Group [*F*(2,67) = 10.92, *p* < 0.001; see **Figure 4**]. In the TD group, CC rates in the Following round were significantly greater than in the Leading [*t*(34) = 5.26, *p* < 0.001] and JI rounds [*t*(34) = 5.00, *p* < 0.001], which were no different from each other [*t*(34) = 0.664, *p* = 0.511]. In the ASD group, there was no significant difference in CC rates between the rounds [Leading–Following, *t*(33) = 0.568, *p* = 0.574; Following-JI, *t*(33) = 0.855, *p* = 0.399; Leading-JI, *t*(33) = 0.133, *p* = 0.895]. *Post hoc* comparisons within each round showed a robust group difference in the Following round [TD > ASD, *t*(67) = 7.96, *p* < 0.001]; but no significant differences in the Leading [*t*(67) = 0.36, *p* = 0.723] and JI rounds [*t*(67) = 0.694, *p* = 0.490].

**FIGURE 5:**
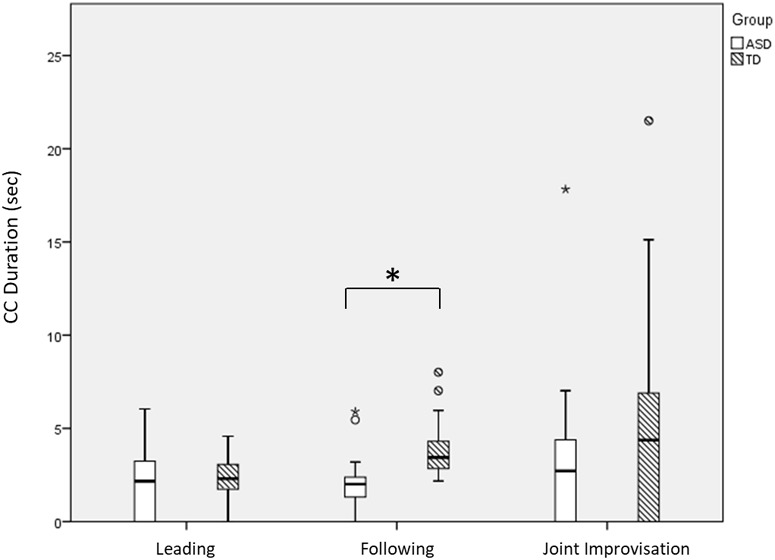
Participants’ average CC duration. When entering CC periods, TD participants maintained them longer than ASD participants (main effect of Group). Large asterisks represent *p* < 0.05 group differences. Boxplots: the solid line represents the median of the distribution; the outlines of the box represent the interquartile range, or 25th–75th percentiles; the whiskers represent the upper and lower quartiles, excluding outliers; small points and asterisks represent outliers and extreme outliers, respectively.

Analysis of CC duration showed that it differed across rounds [*F*(2,130) = 8.24, *p* < 0.001], increasing significantly from Following to Leading [*t*(67) = 3.37, *p* = 0.001], and marginally so from Leading to JI [*t*(66) = 1.92, *p* = 0.06], see **Figure 5**. Notably, CC duration was longer among TD participants compared with ASD participants [main effect of Group: *F*(1,65) = 8.57, *p* = 0.005]. We found no significant Group by Round interaction effect [*F*(2,130) = 2.12, *p* = 0.124], suggesting that ASD participants reached shorter CC moments across all game rounds.

Given the observed dependency between CC and velocity, and the reduced velocity of ASD participants in the Following round, we proceeded with exploratory analyses, asking whether the reduced CC levels exhibited by ASD participants could be explained by their slower motion.

**FIGURE 6:**
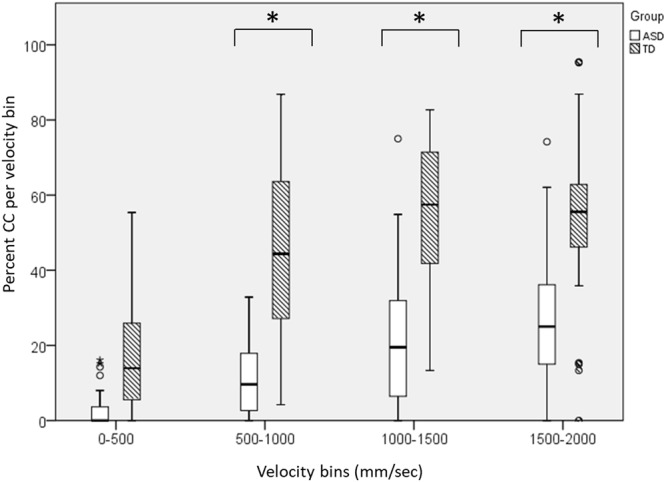
A direct comparison of the %CC segments by velocity bins, between ASD and TD participants, during the Following round. In both groups, %CC increased in higher velocities. Yet even when controlling for different game velocities, TD participants attained higher levels of CC across all velocity intervals. Large asterisks represent *p* < 0.05 group differences. Boxplots: the solid line represents the median of the distribution; the outlines of the box represent the interquartile range, or 25th–75th percentiles; the whiskers represent the upper and lower quartiles, excluding outliers; small points and asterisks represent outliers and extreme outliers, respectively.

To that end, we quantified the % CC in pre-defined velocity bins (thus controlling for velocity) and compared it between groups (see **Figure 6**). First, we replicated the above observed dependency between CC and velocity, as participants’ ability to attain CC increased significantly with velocity range [main effect of Velocity Bin, *F*(3,165) = 49.86, *p* = < 0.001], such that percent CC was lowest in the slowest range, and increased with each velocity range [0–500 v. 500–1000 mm/s, *t*(66) = 8.08, *p* < 0.001; 500–1000 vs. 1000–1500 mm/s, *t*(65) = 4.29, *p* < 0.001], with no significant difference between the fastest ranges, 1000–1500 and 1500–2000 mm/s [*t*(57) = 0.05, *p* = 0.96]. Notably, this pattern was evident both in the ASD and in the TD participants suggesting that the association between CC and velocity was similar in both groups [no effect of Group by Velocity Bin interaction, *F*(3,165) = 2.47, *p* = 0.13]. We also found a main effect of Group [*F*(1,55) = 54.73, *p* = < 0.001] such that TD participants had significantly greater CC within the velocity bins, compared with ASD participants, despite controlling for velocity.

Finally, to determine whether participants’ CC rates were above chance levels, we conducted a pseudo-pairs analysis. When pairing participants’ motor traces to one another randomly, pseudo-CC rates were significantly lower than the actual observed CC for both ASD and TD participants, in all rounds [Mean (SD) of average CC across all rounds in ASD participants was 0.003 (0.008), and in TD participants, 0.003 (0.007); see Supplementary Table 4 for data and results of independent *t*-tests].

To summarize, ASD participants attained CC above chance level, but their percent CC was attenuated when following an expert leader. Furthermore, the duration of their CC segments was shorter, compared with TD participants’, across all rounds. ASD and TD participants had qualitatively similar game patterns, such that the probability of CC increased with velocity; yet ASD participants nevertheless had a lower probability of CC, compared with TD participants, even when controlling for velocity.

### Relating Motor and Social Skills to Performance on the MG

#### Motor Skills

Participants with ASD performed less well than TD participants in the general motor abilities task [PANESS: *t*(66) = 3.19, *p* = 0.002], and the imitation task [*t*(67) = 2.25, *p* = 0.03]; and moved more slowly than TD participants in the Repetitive motion task [*t*(63) = 2.87, *p* = 0.006]. ASD participants performed on par with TD participants on the dyspraxia task [*t*(67) = 1.38, *p* = 0.17] and on the specially designed motor tasks of Reaching accuracy [*t*(64) = 1.23, *p* = 0.221], Proprioceptive accuracy [*t*(56) = 0.713, *p* = 0.479], and Following a Moving Target [*t*(34) = 1.05, *p* = 0.303] (see **Table 3**).

**Table 3:** Participants’ measures in the battery of motor tasks.

Motor task	ASD	TD
Reaching accuracy (distance from target in mm)	18.98 (11.6)	15.9 (7.8)
Proprioceptive accuracy (distance from target in mm)	46.28 (43.6)	40.10 (21.6)
Repetitive motion task (average maximum velocity per segment in mm/s)	1968.2 (812.7)	2520.3 (736.9)
Following a Moving Target (percent CC)	2.2 (4.5)	0.6 (0.9)
PANESS total	–0.98 (0.37)	0.17 (0.33)
Dyspraxia (percent correct)	98 (7.5)	100 (0.0)
Imitation score	7.29 (1.4)	8.14 (1.7)

To determine whether motor skills impacted MG performance, particularly in the Following round, we conducted a negative binomial regression test, with percent CC in the Following round as the dependent variable. We entered Group (ASD and TD) as a categorical predictor, PANESS and speed in the Repetitive task as continuous predictors, and the interaction of the two continuous predictors with Group into the model.

We found a marginally significant simple effect of Group, such that ASD participants had lower CC [*B* = -0.898; *SE* = 0.501; Wald Chi-Square = 2.12; *p* = 0.07; *Exp(B)* = 0.41]; a simple effect of Repetitive [*B* = 0; *SE* = 0.001; Wald Chi-Square = 3.99; *p* = 0.046; *Exp(B)* = 1.00], such that participants who moved faster during the Repetitive motion task, attained more CC in the Following round of the MG. And an interaction effect of Group and PANESS [*B* = 0.87; *SE* = 0.424; Wald Chi-Square = 4.19; *p* = 0.041; *Exp(B)* = 2.38]. We did not find a significant simple effect of PANESS; nor an interaction between Group and Repetitive (see Supplementary Table 5). *Post hoc* analyses revealed that in the ASD group, PANESS significantly correlated with CC in Following, while in the TD group they did not. A full table of correlations can be found in the Supplementary Tables 6 and 7.

In sum, ASD participants performed less well in the general motor task, PANESS, and moved more slowly than TD participants, even when instructed to ‘move as fast as you can’ (in the Repetitive motion task). Yet these basic motor abilities affected each group differently in terms of their ability to attain CC when following an expert. In both groups, faster response to the repetitive motion task predicted more CC in the Following round; while only in the ASD group were general motor skills (PANESS) additionally predictive of CC. Importantly, even while controlling for motor differences, group differences in CC were still apparent; meaning that motor abilities among ASD participants account for some, but not all, of their reduced CC in following.

#### Social Skills

ASD participants performed less well than TD participants on all social measures. Self-report questionnaire data are reported in **Table 1**; conversation ratings are reported in Supplementary Table 8. During the conversation task, ASD participants exhibited fewer looks [*t*(65) = 4.28, *p* < 0.001], fewer headshakes/nods [*t*(65) = 4.24, *p* < 0.001], and fewer smiles [*t*(65) = 4.32, *p* < 0.001]; and were rated lower on affective engagement [*t*(65) = 10.04, *p* < 0.001] and flow [*t*(65) = 4.93, *p* < 0.001]. To determine whether these social skills predicted the percent CC in the Following round, we entered the composite score of conversation skills, and the scores of the four self-report measures (SRS, TAS, TEQ, and RMET) as continuous predictors in a binomial regression model, and tested for the interaction of Group with all these predictors. We found no significant effect of Group [*B* = 0.500; *SE* = 2.16; Wald Chi-Square = 0.05; *p* = 0.81; *Exp(B)* = 1.65]; nor any simple effect of predictors. We found an interaction effect of TAS and Group [*B* = -0.04; *SE* = 0.018; Wald Chi-Square = 5.74; *p* = 0.02; *Exp(B)* = 0.96], such that in the ASD group, greater alexithymia on the TAS correlated with lesser CC in Following (*r* = -0.51, *p* = 0.003); while in the TD group, this relationship was not significant (*r* = -0.03, *p* = 0.87). No other interaction effects were significant (see Supplementary Table 9). A full table of correlations can be found in the Supplementary Tables 10 and 11. In sum, while ASD participants performed more poorly on all social measures, on the whole, these did not significantly predict the ability to attain CC; though higher alexithymia was found to decrease CC among ASD, but not TD, participants.

### Participants’ Experience of the Game

#### Affective Experience of the Game

ASD participants experienced the expert improviser as less responsive to their movements than TD participants (*U* = 184; *p* = 0.022) and were less likely to want to continue playing with them (*U* = 177; *p* = 0.043); but their ratings did not differ in their general affect (How did you feel during the game?); and their perception of how self-centered the other player was (*p*’s>0.17). Among ASD participants, more positive affective ratings of the game correlated with greater %CC in the JI round (ρ = 0.412, *p* = 0.033); and their ratings of how much they would like to continue playing with this partner correlated with their %CC (ρ = 0.426, *p* = 0.027) and CC duration (ρ = 0.384, *p* = 0.048) in the Following round (see Supplementary Table 12).

#### Subjective Experience of Difficulty

**FIGURE 7:**
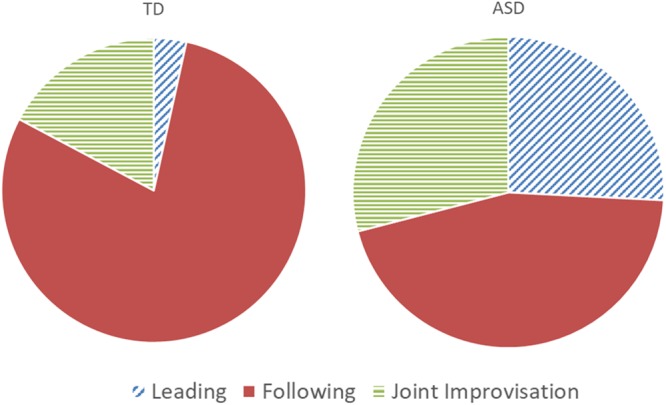
Participants’ ratings of most difficult round. TD participants had significantly different ratings, compared with ASD participants. A majority of TD participants experienced the round in which they followed an expert improviser as most difficult. By contrast, ASD participants were more varied in their ratings of most difficult round, with some experiencing the following round as most difficult, and other experiencing the leading or JI round as most difficult.

TD and ASD participants differed significantly in their ratings of most difficult game round [χ^2^(2,60) = 8.72, *p* = 0.013]: while 74% of TD participants rated the Following round as most difficult, only 45% of ASD participants perceived it as such, with 26% perceiving of the Leading round as most difficult, and 29% perceiving of the JI round as most difficult (see **Figure 7**). The two groups did not differ significantly in their rating of the easiest round [χ^2^(2,62) = 0.924, *p* = 0.630], with the majority of participants in each group rating the Leading round as easiest (52% ASD; 49% TD). Participants’ subjective ratings of hardest round did not relate to %CC in either group, in any of the rounds, as determined by a series of binomial regression tests.

#### Qualitative Descriptions of the Game

As noted above, the majority of both the ASD and TD participants experienced the Following round as most difficult. Participants explained their experience such:

Participants with ASD who experienced the Leading round as most challenging, described their difficulty as stemming from the burden of creating a motion (“I was uncertain about what to do,” “I had to create situations for my partner to follow”), or taking the lead in an interaction (“I’m not used to leading”). By contrast, many TD participants found the Leading round easiest because of their degree of control and hence predictability (“easiest when you’re leading because you know where it’s going”).

ASD participants who experienced the Following round as most challenging, tied their experience to the difficulty in predicting the other player’s motion (“I need to predict the other’s motions and follow them, it’s like they ‘control’ me” “What I predicted didn’t always happen,”), the need to stay focused and respond precisely (“It’s hard to follow,” “I had to stay focused,” “when you follow, you have to always be maximally alert, to capture the other’s motion at the millisecond level”), or their poorer motor ability, compared with the experimenter (“She moved very fast”; “her fine motor skills were better than mine,” “because my response time is slow, it was very hard for me.”). TD participants provided qualitatively similar responses when explaining their difficulty in the Following round.

Finally, ASD Participants who experienced the JI round as most challenging described their confusion in not knowing who is leading (“I didn’t know who’s leading, it was a mishmash,” “There’s no one showing me the way, I have to figure it out on my own”), while some understood the need, and difficulty, of letting go of control in order to enable successful JI (“I didn’t know what rhythm to do; I had to just let it happen”). TD participants who experienced the JI round as most challenging emphasized the problem of role coordination: (“you don’t know if you’re leading or not, so it’s like a mess”; “you realize quickly that someone has to lead”).

In sum, ASD participants did not differ from TD participants in their general affective experience of the game, though they experienced the experimenter as less responsive. Interestingly, among ASD participants, more positive ratings of the game and of their experience with the partner correlated with greater %CC in the Following and JI rounds. ASD participants further reported different challenges arising from each round’s social and motor demands.

## Discussion

This study set out to examine the motion patterns of adults with ASD during an open-ended JI game. Our study presents the first evidence that participants with ASD can attain highly synchronized CC motion while playing with an expert improviser, though their degree of CC is attenuated in several ways.

First, we found that ASD participants had reduced CC compared with TD participants, particularly when following an expert improviser (**Figure 4**). While TD participants had a significant increase in CC when following an expert improviser (compared with when they led or engaged in JI), ASD participants did not demonstrate this increase. Second, even as they entered CC periods, ASD-expert dyads maintained them for shorter periods than TD-expert dyads, meaning they disengaged earlier, in all rounds (**Figure 5**). The fact that ASD participants had the most difficulty in the Following round aligns with the vast literature on imitation deficits in ASD (Edwards, 2014; Vivanti and Hamilton, 2014), and more recently, with accounts of reduced synchronization in youth with ASD during rhythmic, repetitive tasks (Marsh et al., 2013; Fitzpatrick et al., 2016; Romero et al., 2016).

Analysis of basic motor characteristics during the MG showed that ASD participants’ motion was no less complex than TD participants’ in all rounds (**Figure 3A**). In other words, contrary to our hypothesis, they were not limited to repetitive strokes during the game, suggesting spared improvisational ability among ASD participants. By contrast, the velocity of their motion during the Following round was significantly reduced compared to TD participants. Building upon the extensive motor measurement, enabled by the MG experimental platform, we conducted an in-depth analysis of the relation between CC and velocity in the TD and ASD groups. We found that both groups exhibited a similar general pattern, such that CC levels increased as motion velocity increased, in line with previous findings in the MG (Noy et al., 2017). The similarity of this velocity-CC association across the two groups further supports the suggestion that ASD participants’ general game pattern was similar to TD participants’. Of note, the experimenter, an expert improviser with experience working with people with ASD, who was not blind to participants’ diagnosis, moved more slowly when leading the ASD participants than the TD participants.^3^ Thus, it is possible that the fact that ASD participants were not exposed to higher velocities may have impeded their ability to attain CC. And yet, as the velocity bins analysis demonstrates, the level of CC among ASD participants was reduced compared to TDs, even when controlling for velocity (**Figure 6**). In other words, even when ASD participants moved at the same velocity as TDs, they still showed reduced levels of CC. Taken together, these findings suggest that while ASD participants’ performance was characteristic of a dyadic improvisation game, they could not attain comparable levels of motor synchrony as TD participants when following an expert improviser and this difference could not be explained by differences in velocities between the two groups.

An examination of ASD participants’ abilities in individual motor tasks showed a generally reduced motor performance in this group. These individual motor tasks had different predictive effects on the dyadic CC measure. Specifically, ASD participants moved more slowly than TD participants when asked to ‘move as fast as you can’ in the Repetitive Motion task on the MG console; and performance in this task predicted CC levels in the MG in both groups. At the same time, ASD participants showed reduced general motor performance (PANESS) compared to TD participants, which predicted their level of CC in Following, but did not predict levels of CC in the TD group. In sum, individual-level motor difficulties among ASD participants, explained some, but not all, of the group differences found in the degree of dyadic-level CC.

Notably, ASD participants were capable of creating motions which led to comparable levels of synchronization with an expert, as in TD participants, in the Leading round. This finding could be explained by the fact that the burden of synchronization lay mostly on the expert improviser, which skillfully synchronized with both groups of participants; or that ASD participants simply have no difficulty leading an interaction into a CC state (in line Koehne et al., 2016b, who found that ASD and TD participants reported no difference in perceived synchrony when leading a repetitive synchronization task). More remarkably, ASD participants did not differ, at the outset, from TD participants in levels of CC during the JI round, which presumably was most difficult to coordinate (Noy et al., 2011). It is possible, as reported by the experimenter, that despite the instruction to move together during the JI round, players in fact took turns leading and following, with participants taking the lead the majority of the time; such that in many instances the JI round became similar to the Leading round.

Our findings extend prior research on JI, and align with results regarding the game patterns of novices playing with an expert improviser (Noy et al., 2011; Feniger-Schaal et al., 2016). First, our results align well with previous data collected in a similar setup, in which novice TD players playing against an expert improviser showed similar rates of CC and complexity scores as our participants (Feniger-Schaal et al., 2016; see Supplementary Table 1). Moreover, as in our study, novices show less CC and slower velocities in JI, compared with Leading/Following rounds, presumably because they are trying harder to synchronize with one another, while expert improvisers show the opposite pattern, presumably because they are more comfortable in JI. At the same time, similarly to experts, our participants show an increase in CC duration for JI rounds, compared with Leading/Following rounds, presumably because this round is more conducive to longer CC periods. Together, these results begin to trace a continuum of JI abilities – from highly specialized experts, through novices, and individuals with social impairments.

Interestingly, we found that lesser alexithymia predicted greater CC in Following among ASD participants. It has been argued that alexithymia, or the inability to identify and describe one’s own emotions, also subserves the ability to empathize with others (Bird and Cook, 2013). Thus, participants who were less capable of perceiving of their own emotions, may have had more difficulty following another player’s motions.

As for the other social-emotional measures collected in this study, we did not find a significant association between them and the performance in MG. It is possible that social skills and synchronization skills pertain to two separate faculties, and that while ASD participants have most of the necessary skills for sensory-motor synchronization, performing a successful social interaction requires a different set of skills, in which they are impaired. Alternatively, it is possible that our sample size was not large enough to detect correlations, or that our social measures were not sensitive enough to skills that would be correlated with performance on the MG. Notably, Koehne et al. (2016b) related performance on a repetitive synchronization tasks with social measures of cognitive empathy (RMET and the Movie for the Assessment of Social Cognition; Dziobek et al., 2006), and found that among ASD participants, cognitive empathy correlated with one condition in one of their synchronization tasks (following a putative human), but did not find significant correlations during other conditions. Further research is needed in order to determine whether sensory-motor synchronization is indeed related to social skills in individuals with ASD, or whether this relationship is limited to specific situations.

Participants’ reports on their experience of the MG provide further insight onto their affective state during the game. Participants with ASD did not differ from TD participants in their affective experience of the game, and in their perception of the experimenter’s self-centeredness, but ASD participants were less likely to want to continue playing, and also experienced the expert as less responsive to their movements. It is possible that ASD participants’ reports were expressions of a genuine social difficulty in perceiving the experimenter’s responsiveness, and hence in their desire to play. Concurrently, it is possible that ASD participants cared less for social desirability than TD participants did (meaning that TD participants’ ratings were inflated). Regardless, among ASD participants, positive affect and a desire to continue playing with the experimenter correlated significantly with their average %CC. The relationship between experience and CC could be a bi-directional one: participants who experienced CC could be more likely to report on positive experiences of the game (van Baaren et al., 2004; Valdesolo and Desteno, 2011), or the fact that they experienced the game as positive, may have led them to greater coordination with their partner (Chartrand and Lakin, 2013). Further research is needed in order to disentangle this causal relationship.

Surprisingly, though ASD participants performed significantly less well than TD participants in the Following round (as measured by %CC), the majority of TD participants reported that the Following round was most difficult for them, whereas ASD participants were more evenly distributed in their experience of most difficult round. Though these ratings did not relate directly to participants’ performance, they nevertheless provide a view onto participants’ experiences. Notably, ASD participants reported various challenges, in all three rounds. The Leading round was challenging due to the need to create motions and lead the other player, which relates to ASD individuals’ known challenge with creativity and improvisation (Lord et al., 2012). In the Following round, ASD participants reported the challenge of poorer motor abilities, difficulty in prediction, and difficulty in sustaining focus, all known as hurdles for individuals with ASD (Bhat et al., 2011; Sinha et al., 2014; Chita-Tegmark, 2016). Finally, in JI, ASD participants reported a difficulty in coordinating leadership and ‘letting go,’ which are indeed cornerstones of JI (Schechner, 1994). Together, these reports point to the high levels of self-awareness among our high-functioning ASD participants, and at the same time, the heterogeneity of their challenges. Interventions aiming to increase synchronization in individuals with ASD must take heed of these challenges, and aid participants according to their needs.

In sum, participants with ASD were capable of attaining CC during joint synchronization, but their performance was attenuated in several ways, including a reduced % CC, particularly in the Following round, and reduced CC duration throughout the game. We have shown that motor skills, and the subtle ability to modulate motion velocity, may account for some of these differences. Our data further suggest that alexithymia impedes ASD participants from following an experimenter’s motions. Yet participants’ reports suggest that other cognitive and emotional factors, which were not directly tested, may be affecting their performance. Successful joint synchronization requires sustained attention, motivation, affective enjoyment, and arguably, an ability to detect those special moments of CC, and a tendency to find them rewarding; all of which may be reduced in autism. Moreover, individuals with ASD may find the social situation and open-ended nature of the MG anxiety-provoking. As we have seen, ASD participants’ positive affect correlates with their average CC, meaning their performance on the MG may be impacted by their degree of social motivation, or the degree to which they find synchronization rewarding. Individuals with ASD are known to be less motivated in social situations (Chevallier et al., 2013), and to find them less rewarding (Scott-Van Zeeland et al., 2010; Dichter et al., 2012). Thus, it is possible that participants with ASD had difficulty detecting that they were *in* a CC moment; or perhaps, the CC moments did not elicit the same sense of ‘togetherness’ experienced by expert improvisers (Noy et al., 2015b). Future research should continue to examine the experience of individuals with ASD during JI, in order to disentangle the effects of motivation, perception, and reward, in supporting joint synchronization. Furthermore, future research should continue to elucidate the neurocognitive bases involved in the MG, disentangling those from similar functions, such as imitation and mimicry (Vivanti and Hamilton, 2014).

Our study had several limitations. First, by nature of the fact that participants played against a human player in a dyadic interaction, each participant played a slightly different variant of the game. Furthermore, the experimenter was aware of participants’ diagnostic status, and this impacted her movement velocity. In order to better detect group differences in performance, computer-controlled versions of the MG could be used (Noy et al., 2017; Słowiñski et al., 2017). Inversely, as we sought to provide ASD participants with the best conditions to attain CC, and hence set them to play with an expert improviser, it is possible that their improvisational dynamics would be different in a more naturalistic dyad, such as with an ASD or TD peer, or with a close caregiver. Very little is known about the quality of interactions between two individuals with autism, as the majority of the field has focused on interactions between an individuals with autism and their neurotypical caregivers or peers (Arciuli and Brock, 2014). Given their difficulties in social interaction, it is possible that ASD–ASD interactions would be further impaired, or in the MG, less synchronized. Yet, ethnographic evidence suggests that individuals with autism can create their own unique, creative, interactions when engaging with one another (Fein, 2015), and interactions between a child with ASD and their friend have been shown to have greater conversational and pragmatic quality than with a non-friend (Bauminger-Zviely et al., 2014). Future research should examine the variance of ASD participants’ improvisational patterns with different game partners, of varying levels of proximity.

The fact that we did not find significant correlations between our MG measures and social skills may suggest that our social tasks and questionnaires were not sensitive enough. Future research should continue to investigate the effects of social motivation, social anxiety, attention, and reward on JI, and social interaction more broadly, among individuals with ASD. A further limitation of our study is the sparse measurement of intelligence levels, using only two sub-test of the WASI as indicators of IQ, and the fact that TD participants had more years of education, and marginally greater Performance IQ than ASD participants. Of note, we entered IQ and years of education as covariates in all of our analyses as a preliminary check, and did not find any difference in the pattern of results; and further did not find any correlation between IQ and MG measures, suggesting that MG performance is not related to IQ. Nevertheless, future studies should continue to collect data from a wider range of ASD participants, of both low and high levels of functioning, alongside well-matched TD samples, in order to better characterize the relationship between intelligence, motor abilities, and JI. Similarly, our sample was not large enough to parse between sub-groups of individuals with autism who may have comorbid disorders, such as anxiety, depression, or schizophrenia; conditions that may significantly affect MG performance. Future studies should examine the possible effects of comorbid conditions in ASD on MG performance, and JI more broadly.

## Conclusion

In an open-ended, JI game, adults with ASD were capable of attaining highly synchronized motions when playing with an expert improviser; though their synchronization was attenuated in several ways. While motor modulation abilities may account for part of this attenuation, unexplored social and emotional factors may also impact their performance. On the whole, it is important to remember that joint synchronization was not inexistent, but simply reduced, in individuals with autism; and their existing synchronization skills may be further harnessed in clinical settings (Koehne et al., 2016a), to support their personal expression and social rapport.

## Author Contributions

R-SB: Study design, data collection, data analysis, data interpretation, and write up; LN: Study design, data interpretation, and write up; TA: Data analysis and data interpretation; RG: Data analysis; RC: Data analysis; YG: Study design, data interpretation, and write up; NL-B: Study design and data interpretation.

## Conflict of Interest Statement

The authors declare that the research was conducted in the absence of any commercial or financial relationships that could be construed as a potential conflict of interest.

## Footnotes

1 The complexity measure is similar to the wavelet-compression-ratio measure described in Noy et al. (2011; see Supplementary Information Figures 5, 6). In brief, a position vector of one player (the original signal) is decomposed into small building blocks of varying size from a specific family of shapes (called wavelets). Using these building blocks, the original signal can be reconstructed (with some small error) into a much smaller reconstructed signal. The reconstruction thus compresses the original signal. The compression ratio is the ratio between the length of the compressed signal and the length of the original signal. A simpler signal can be reconstructed with fewer elements, and will haves a smaller compression ratio. This measure is based on the concept of Kolmogorov complexity, where the complexity of a signal is defined by the minimum length of its description. To set the length of the compressed signal the algorithm pre-defines an allowed error. In the current version, the algorithm searches for the minimal set of wavelets that successfully captures 90% of the energy (sum-of-squares) of the original signal. The algorithm is publically available here: https://figshare.com/articles/Mirror_Game_Wavelet-Complexity_Measure/5372677.

2 The algorithm is publicly available here: https://figshare.com/articles/Mirror_Game_-_CC_Measure/5373157.

3 This, in itself, is not surprising, given that the MG is a dyadic, interactive game. According to the experimenter’s report, she often found that ASD participants led the first round very quickly, and wanted to show them that is possible to move more slowly; while TD participants often began by leading slowly and hesitantly, and so the experimenter tried to ‘shake them out’ into more rapid movements. Of course it is also possible that she subconsciously slowed her movements when playing with ASD participants, to better accommodate them. These kinds of bi-directional effects are part and parcel of this dyadic game, and can only be disentangled if using a non-human game partner.

## Abbreviations

ADOS: Autism Diagnostic Observation Schedule
ASD: autism spectrum disorder
CC: co-confident
JI: joint improvisation
MG: mirror game
PANESS: The Revised Neurological Examination for Subtle Signs
RMET: Reading the Mind in the Eyes
SRS: Social Responsiveness Scale
TAS: Toronto Alexithymia Scale
TD: typically developing
TEQ: Toronto Empathy Questionnaire
WASI: Wechsler Abbreviated Scale of Intelligence
