# Supplementary material for: Patterns of Joint Improvisation in Adults with Autism Spectrum Disorder
Source: Front Psychol. 2017 Oct 24;8:1790. doi: 10.3389/fpsyg.2017.01790 (PMC5660713; doi:10.3389/fpsyg.2017.01790)
Supplement: Supplementary file 1 [file Table_1.docx]

Supplementary Material

**Patterns of Joint Improvisation in Adults with Autism Spectrum Disorder**

**Rachel-Shlomit Brezis*, Lior Noy, Tali Alony, Rachel Gotlieb, Rachel Cohen, Yulia Golland & Nava Levit-Binnun**

*** Correspondence:** Rachel-Shlomit Brezis: brezisrs@gmail.com

# Supplementary Data

## Introduction: MG motor measures collected in prior studies with a similar setup: TD novices playing against an expert improviser (Feniger-Schaal et al., 2015)

| **Motor measure** | **MG round** | **TD novices Mean(SD)** |
| --- | --- | --- |
| **Complexity**  **(Wavelets decomposition compression ratio)** | Leading | 0.025 (0.012) |
|  | Following | 0.019 (0.005) |
|  | Joint Improvisation | 0.019 (0.009) |
| **Percent Co-Confident (CC) Motion** | Leading | 6.3 (10.1) |
|  | Following | 9.8 (5.9) |
|  | Joint Improvisation | 9.4 (9.8) |

Table 1: Motor measures in the Mirror Game collected from novice-expert dyads (*n*=48) in a similar experimental setup (see Feniger-Schaal et al., 2015). These data serve as a comparison basis for our data.

## Post-game questionnaire

1. How did you feel during the game? (5-point emotional slider from big smile=1 to big frown=5)
2. The game had three rounds: a round in which you were the leader, a round in which you were the follower, and a round in which there was no defined leader/follower.
   1. Which round was easiest for you?
   2. Which round was hardest?
3. During the part where no leader/follower was defined, did you feel one of the players was leading? (No, I felt that we were leading together/ Yes, I felt that I was leading more/ Yes, I felt that the other player was leading more)
4. Did you feel that the other player responded to your movements? (1=Not at all - 5=Very much)
5. Did you feel that the other player was self-absorbed? (1=Not at all - 5=Very much)
6. Would you like to continue playing with this player? (1=Not at all - 5=Very much)
7. Did the game remind you of anything from your daily life?

## Analysis: Criteria for determining co-confident motion (CC)


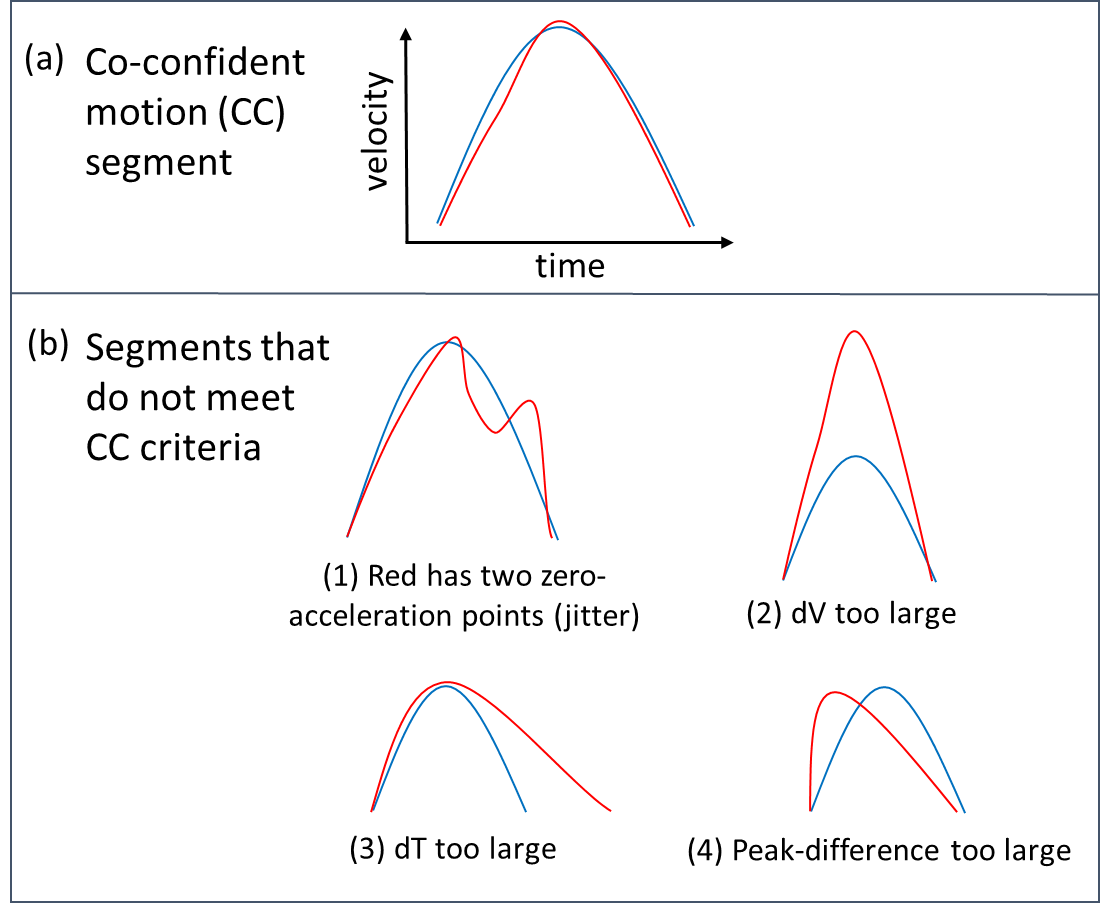


Figure 1: Criteria for determining Co-confident motion (CC) in the mirror game. (a) A segment is deemed as “Co-confident motion” when Red and Blue players both have a smooth motion single zero-acceleration crossing (that is, a single velocity peak, with no jitter), and their motions are similar. (b) Segments are deemed as “not-CC” if they meet one or more of the following conditions: (1) there are more than one acceleration zero-crossings (‘jitter’); (2) the normalized velocity error (dV) between the two segments is greater than 0.95; (3) the temporal distances of their stopping point events (dT) are longer than 0.09 sec; (4) the difference in velocity peaks is greater than 0.3.

## Preliminary analyses: correlations of IQ with measures of CC

### ASD

|  | | % CC Leading | % CC Following | % CC JI | PIQ | VIQ |
| --- | --- | --- | --- | --- | --- | --- |
| % CC Leading | *r* | 1 | .004 | -.087 | .031 | -.212 |
|  | *p* |  | .983 | .626 | .862 | .245 |
| % CC Following | *r* | .004 | 1 | .117 | .149 | .014 |
|  | *p* | .983 |  | .508 | .406 | .941 |
| % CC JI | *r* | -.087 | .117 | 1 | .017 | -.029 |
|  | *p* | .626 | .508 |  | .924 | .875 |
| PIQ | *r* | .031 | .149 | .017 | 1 | .430^*^ |
|  | *p* | .862 | .406 | .924 |  | .014 |
| VIQ | *r* | -.212 | .014 | -.029 | .430^*^ | 1 |
|  | *p* | .245 | .941 | .875 | .014 |  |

Table 2: Table of correlations between measures of CC in the Mirror Game and IQ in the ASD group. * Correlation is significant at the 0.05 level.

### TD

|  | | % CC Leading | % CC Following | % CC JI | PIQ | VIQ |
| --- | --- | --- | --- | --- | --- | --- |
| % CC Leading | *r* | 1 | .034 | .477^**^ | .031 | -.144 |
|  | *p* |  | .844 | .004 | .861 | .465 |
| % CC Following | *r* | .034 | 1 | .234 | .113 | .116 |
|  | *p* | .844 |  | .177 | .525 | .557 |
| % CC JI | *r* | .477^**^ | .234 | 1 | .090 | -.203 |
|  | *p* | .004 | .177 |  | .613 | .300 |
| PIQ | *r* | .031 | .113 | .090 | 1 | .067 |
|  | *p* | .861 | .525 | .613 |  | .735 |
| VIQ | *r* | -.144 | .116 | -.203 | .067 | 1 |
|  | *p* | .465 | .557 | .300 | .735 |  |

Table 3: Table of correlations between measures of CC in the Mirror Game and IQ in the TD group. ** Correlation is significant at the 0.01 level.

## Pseudo-pairs analysis

|  | TD | | | ASD | | |
| --- | --- | --- | --- | --- | --- | --- |
|  | Actual pairs Mean (SD) | Pseudo-pairs  Mean (SD) | Statistic | Actual pairs Mean (SD) | Pseudo-pairs Mean (SD) | Statistic |
| Leader | 10.9 (12.8) | 0.25 (0.63) | *t*=20.3, *p*<.001 | 9.8 (14.7) | 0.28 (0.80) | *t*=15.2, *p*<.001 |
| Follower | 25.7(10.9) | 0.68 (0.90) | *t*=51.2, *p*<.001 | 8.2 (6.7) | 0.67 (0.90) | *t*=23.7, *p*<.001 |
| Joint Improvisation | 12.5(13.9) | 0.06 (0.33) | *t*=21.3, *p*<.001 | 10.3 (13.0) | 0.06 (0.28) | *t=*18.3, *p*<.001 |
| All rounds | 16.4 (14.1) | 0.33 (0.71) | *t*=46.3, *p*<.001 | 9.4 (11.8) | 0.34 (0.75) | *t*=30.3, *p*<.001 |

Table 4: Results of a pseudo-pairs analysis of CC rates, in which participants were paired to other participants, rather than to their actual game partner (an expert improviser). This analysis was conducted to determine chance levels of CC in ASD and TD groups. Each participant was paired to all other participants in the group, in turn, resulting in 1683 pseudo-pairs in the TD group, and 1785 pseudo-pairs in the ASD group. We further include here a comparison with the actual pairs data, and results of the independent t-tests between actual and pseudo-pairs.

## Binomial Regression for Motor Skills on CC

|  | **B** | **SE (B)** | **95% Wald Confidence Interval** | | **Hypothesis test** | | | **Exp(B)** |
| --- | --- | --- | --- | --- | --- | --- | --- | --- |
|  |  |  | **Lower** | **Upper** | **Wald Chi-Square** | **df** | **Sig** |  |
| **(Intercept)** | 2.494 | .3884 | 1.732 | 3.255 | 41.225 | 1 | .000 | 12.104 |
| **Group** | -.898 | .5009 | -1.880 | .083 | 3.217 | 1 | .073 | .407 |
| **PANESS** | -.111 | .2684 | -.637 | .415 | .172 | 1 | .679 | .895 |
| **Repetitive** | .000 | .0001 | .00005 | .001 | 3.999 | 1 | .046 | 1.000 |
| **Group * PANESS** | .868 | .4243 | .037 | 1.700 | 4.190 | 1 | .041 | 2.383 |
| **Group * Repetitive** | -.0002 | .0002 | .000 | .000 | .013 | 1 | .908 | 1.000 |

Table 5: Binomial regression model, testing the impact of Motor skills on MG performance (%CC) in the Following round.

## Correlations: CC and Motor Skills

### ASD

|  | | %CC Leading | % CC Following | % CC JI | Reaching | Proprioception | Repetitive motion | Following a moving target | PANESS | Dyspraxia | Imitation |
| --- | --- | --- | --- | --- | --- | --- | --- | --- | --- | --- | --- |
| %CC Leading | *r* | 1 | .004 | -.087 | .002 | -.183 | -.022 | .099 | -.218 | .111 | -.059 |
|  | *p* |  | .983 | .626 | .990 | .392 | .904 | .624 | .216 | .530 | .742 |
| % CC Following | *r* | .004 | 1 | .117 | -.446^*^ | -.115 | .278 | .065 | .331 | .151 | -.047 |
|  | *p* | .983 |  | .508 | .012 | .592 | .117 | .747 | .055 | .394 | .794 |
| % CC JI | *r* | -.087 | .117 | 1 | -.189 | -.175 | .055 | .138 | .071 | .127 | .030 |
|  | *p* | .626 | .508 |  | .308 | .413 | .762 | .492 | .690 | .475 | .865 |
| Reaching | *r* | .002 | -.446^*^ | -.189 | 1 | -.025 | -.514^**^ | .487^*^ | -.291 | -.042 | -.271 |
|  | *p* | .990 | .012 | .308 |  | .911 | .004 | .012 | .112 | .823 | .141 |
| Proprioception | *r* | -.183 | -.115 | -.175 | -.025 | 1 | .166 | -.046 | .083 | -.173 | .300 |
|  | *p* | .392 | .592 | .413 | .911 |  | .437 | .856 | .701 | .420 | .154 |
| Repetitive motion | *r* | -.022 | .278 | .055 | -.514^**^ | .166 | 1 | -.148 | .086 | .090 | .215 |
|  | *p* | .904 | .117 | .762 | .004 | .437 |  | .471 | .633 | .616 | .229 |
| Following a moving target | *r* | .099 | .065 | .138 | .487^*^ | -.046 | -.148 | 1 | -.064 | .094 | -.044 |
|  | *p* | .624 | .747 | .492 | .012 | .856 | .471 |  | .751 | .642 | .829 |
| PANESS | *r* | -.218 | .331 | .071 | -.291 | .083 | .086 | -.064 | 1 | .175 | .176 |
|  | *p* | .216 | .055 | .690 | .112 | .701 | .633 | .751 |  | .321 | .320 |
| Dyspraxia | *r* | .111 | .151 | .127 | -.042 | -.173 | .090 | .094 | .175 | 1 | .229 |
|  | *p* | .530 | .394 | .475 | .823 | .420 | .616 | .642 | .321 |  | .192 |
| Imitation | *r* | -.059 | -.047 | .030 | -.271 | .300 | .215 | -.044 | .176 | .229 | 1 |
|  | *p* | .742 | .794 | .865 | .141 | .154 | .229 | .829 | .320 | .192 |  |

Table 6: Correlations between CC and Motor skills in the ASD group. * Correlation is significant at the 0.05 level. ** Correlation is significant at the 0.01 level

### TD

|  | | %CC Leading | % CC Following | % CC JI | Reaching | Proprioception | Repetitive motion | Following a moving target | PANESS | Imitation |
| --- | --- | --- | --- | --- | --- | --- | --- | --- | --- | --- |
| %CC Leading | *r* | 1 | .034 | .477^**^ | .109 | .282 | -.091 | -.383 | -.270 | .101 |
|  | *p* |  | .844 | .004 | .531 | .107 | .619 | .309 | .122 | .562 |
| % CC Following | *r* | .034 | 1 | .234 | -.110 | .284 | .464^**^ | -.290 | -.132 | .039 |
|  | *p* | .844 |  | .177 | .529 | .104 | .007 | .449 | .457 | .826 |
| % CC JI | *r* | .477^**^ | .234 | 1 | -.039 | .235 | .179 | -.482 | -.146 | .061 |
|  | *p* | .004 | .177 |  | .822 | .181 | .327 | .189 | .409 | .728 |
| Reaching | *r* | .109 | -.110 | -.039 | 1 | .263 | .186 | .090 | .252 | .219 |
|  | *p* | .531 | .529 | .822 |  | .133 | .308 | .818 | .151 | .206 |
| Proprioception | *r* | .282 | .284 | .235 | .263 | 1 | .115 | .012 | -.203 | .223 |
|  | *p* | .107 | .104 | .181 | .133 |  | .530 | .976 | .257 | .205 |
| Repetitive motion | *r* | -.091 | .464^**^ | .179 | .186 | .115 | 1 | -.718^*^ | .012 | .071 |
|  | *p* | .619 | .007 | .327 | .308 | .530 |  | .029 | .949 | .698 |
| Following a moving target | *r* | -.383 | -.290 | -.482 | .090 | .012 | -.718^*^ | 1 | .399 | .176 |
|  | *p* | .309 | .449 | .189 | .818 | .976 | .029 |  | .327 | .650 |
| PANESS | *r* | -.270 | -.132 | -.146 | .252 | -.203 | .012 | .399 | 1 | -.420^*^ |
|  | *p* | .122 | .457 | .409 | .151 | .257 | .949 | .327 |  | .013 |
| Imitation | *r* | .101 | .039 | .061 | .219 | .223 | .071 | .176 | -.420^*^ | 1 |
|  | *p* | .562 | .826 | .728 | .206 | .205 | .698 | .650 | .013 |  |

Table 7: Correlations between CC and Motor skills in the TD group. * Correlation is significant at the 0.05 level. ** Correlation is significant at the 0.01 level. Note: Correlations of the Dyspraxia score in the TD group could not be computed due to a strong ceiling effect, such that none of the participants made any error on the task, and no variance could be computed.

## Participants’ Social skills, as rated on conversation task

|  | **ASD** | **TD** |
| --- | --- | --- |
| **Looks (count)** | 151.7 (73.8) | 219.8 (56.1) |
| **Headshakes/Nods (count)** | 17.8 (16.1) | 33.5 (14.1) |
| **Smiles (count)** | 4.6 (4.7) | 13.4 (6.2) |
| **Conversation skills composite score** | -.46 (.61) | .57 (.45) |
| **Affective engagement (global rating)** | 2.5 (1.3) | 4.7 (0.5) |
| **Flow (global rating)** | 2.5 (1.2) | 4.7 (0.4) |

Table 8: Participants’ scores on videotaped conversation task, as rated by two independent raters, blind to diagnosis.

## Binomial Regression for Social Skills on CC

|  | **B** | **SE (B)** | **95% Wald Confidence Interval** | | **Hypothesis Test** | | | **Exp(B)** |
| --- | --- | --- | --- | --- | --- | --- | --- | --- |
|  |  |  | **Lower** | **Upper** | **Wald Chi-Square** | **df** | **Sig.** |  |
| **(Intercept)** | 3.142 | 1.7108 | -.211 | 6.495 | 3.372 | 1 | .066 | 23.140 |
| **ASD** | .502 | 2.1592 | -3.730 | 4.734 | .054 | 1 | .816 | 1.653 |
| **TD** | 0^§^ | . | . | . | . | . | . | 1 |
| **Conversation composite** | .236 | .2549 | -.263 | .736 | .858 | 1 | .354 | 1.266 |
| **SRS** | -.009 | .0179 | -.044 | .026 | .243 | 1 | .622 | .991 |
| **TAS_total** | .001 | .0127 | -.024 | .026 | .006 | 1 | .938 | 1.001 |
| **Total_TES** | -.010 | .0225 | -.054 | .034 | .206 | 1 | .650 | .990 |
| **Total_RMET** | .034 | .0236 | -.013 | .080 | 2.040 | 1 | .153 | 1.034 |
| **ASD * Conversation_composite** | -.149 | .3312 | -.798 | .500 | .202 | 1 | .653 | .862 |
| **TD * Conversation_composite** | 0^§^ | . | . | . | . | . | . | 1 |
| **ASD * SRS** | .014 | .0250 | -.035 | .063 | .334 | 1 | .563 | 1.015 |
| **TD * SRS** | 0^§^ | . | . | . | . | . | . | 1 |
| **ASD * TAS** | -.044 | .0183 | -.080 | -.008 | 5.744 | 1 | .017 | .957 |
| **TD * TAS** | 0^§^ | . | . | . | . | . | . | 1 |
| **ASD * TES** | -.006 | .0293 | -.063 | .052 | .036 | 1 | .849 | .994 |
| **TD * TES** | 0^§^ | . | . | . | . | . | . | 1 |
| **ASD * RMET** | .005 | .0371 | -.068 | .077 | .016 | 1 | .901 | 1.005 |
| **TD * RMET** | 0^§^ | . | . | . | . | . | . | 1 |

Table 9: Binomial regression model, testing the impact of Social skills on MG performance (%CC) in the Following round. ^§^ Set to zero because this parameter is redundant.

## Correlations: CC and Social Skills

### ASD

|  | | %CC Leading | % CC Following | % CC JI | SRS | TAS | TES | RMET | Conversation task composite |
| --- | --- | --- | --- | --- | --- | --- | --- | --- | --- |
| %CC Leading | *r* | 1 | .004 | -.087 | -.106 | .227 | -.039 | -.164 | .012 |
|  | *p* |  | .983 | .626 | .558 | .212 | .827 | .353 | .947 |
| % CC Following | *r* | .004 | 1 | .117 | -.268 | -.491^**^ | .008 | .020 | .068 |
|  | *p* | .983 |  | .508 | .132 | .004 | .965 | .910 | .712 |
| % CC JI | *r* | -.087 | .117 | 1 | -.079 | -.039 | .094 | -.022 | .026 |
|  | *p* | .626 | .508 |  | .664 | .833 | .598 | .902 | .886 |
| SRS | *r* | -.106 | -.268 | -.079 | 1 | .604^**^ | -.061 | .081 | -.227 |
|  | *p* | .558 | .132 | .664 |  | .000 | .734 | .653 | .219 |
| TAS | *r* | .227 | -.491^**^ | -.039 | .604^**^ | 1 | -.144 | -.127 | -.172 |
|  | *p* | .212 | .004 | .833 | .000 |  | .432 | .489 | .364 |
| TES | *r* | -.039 | .008 | .094 | -.061 | -.144 | 1 | .419^*^ | .141 |
|  | *p* | .827 | .965 | .598 | .734 | .432 |  | .014 | .442 |
| RMET | *r* | -.164 | .020 | -.022 | .081 | -.127 | .419^*^ | 1 | .034 |
|  | *p* | .353 | .910 | .902 | .653 | .489 | .014 |  | .855 |
| Conversation task composite | *r* | .012 | .068 | .026 | -.227 | -.172 | .141 | .034 | 1 |
|  | *p* | .947 | .712 | .886 | .219 | .364 | .442 | .855 |  |

Table 10: Correlations between CC and Social skills in the ASD group. * Correlation is significant at the 0.05 level. ** Correlation is significant at the 0.01 level.

### TD

|  | | %CC Leading | % CC Following | % CC JI | SRS | TAS | TES | RMET | Conversation task composite |
| --- | --- | --- | --- | --- | --- | --- | --- | --- | --- |
| %CC Leading | *r* | 1 | .034 | .477** | .178 | .228 | -.085 | -.017 | -.042 |
|  | *p* |  | .844 | .004 | .306 | .188 | .628 | .925 | .809 |
| % CC Following | *r* | .034 | 1 | .234 | .006 | -.034 | .035 | .294 | .195 |
|  | *p* | .844 |  | .177 | .972 | .846 | .843 | .087 | .261 |
| % CC JI | *r* | .477** | .234 | 1 | -.122 | -.042 | .248 | .140 | -.027 |
|  | *p* | .004 | .177 |  | .485 | .810 | .151 | .423 | .878 |
| SRS | *r* | .178 | .006 | -.122 | 1 | .569** | -.521** | .115 | .340* |
|  | *p* | .306 | .972 | .485 |  | .000 | .001 | .509 | .046 |
| TAS | *r* | .228 | -.034 | -.042 | .569** | 1 | -.619** | -.108 | .071 |
|  | *p* | .188 | .846 | .810 | .000 |  | .000 | .536 | .686 |
| TES | *r* | -.085 | .035 | .248 | -.521** | -.619** | 1 | .154 | .002 |
|  | *p* | .628 | .843 | .151 | .001 | .000 |  | .379 | .993 |
| RMET | *r* | -.017 | .294 | .140 | .115 | -.108 | .154 | 1 | .157 |
|  | *p* | .925 | .087 | .423 | .509 | .536 | .379 |  | .369 |
| Conversation task composite | *r* | -.042 | .195 | -.027 | .340* | .071 | .002 | .157 | 1 |
|  | *p* | .809 | .261 | .878 | .046 | .686 | .993 | .369 |  |

Table 11: Correlations between CC and Social skills in the TD group. * Correlation is significant at the 0.05 level. ** Correlation is significant at the 0.01 level.

## Post-game questionnaire ratings

|  | **ASD** | **TD** |
| --- | --- | --- |
| **How did you feel during the game?** | 3.63 (.79) | 4.0 (1.0) |
| **Did you feel that the other player responded to your movements?** | 3.78 (.57) | 4.44 (.72) |
| **Did you feel that the other player was self-absorbed?** | 2.56 (1.18) | 1.89 (.78) |
| **Would you like to continue playing with this player?** | 3.37 (.84) | 4.11 (.78) |

Table 12: Participants’ affective experience of the game, as reported on the post-game questionnaire. Mean(SD).
